# Effectiveness of antepartum breathing exercises on the outcome of labour: A randomized controlled trial

**DOI:** 10.12688/f1000research.75960.3

**Published:** 2023-05-30

**Authors:** Sushmitha R Karkada, Judith A Noronha, Shashikala K Bhat, Parvati Bhat, Baby S Nayak

**Affiliations:** 1 Manipal College of Nursing, Manipal Academy of Higher Education, Manipal, Karnataka, 576104, India; 2 Melaka Manipal Medical College & Dr. TMA Pai Hospital Udupi,, Manipal Academy of Higher Education, Manipal, Karnataka, 576104, India

**Keywords:** Breathing exercises, antenatal, pregnancy childbirth, labour, complementary alternative therapies, Lamaze breathing, natural birth, spontaneous labour

## Abstract

Abstract

Background

Childbirth is a life-transforming intense event to a woman and her family. Even though a variety of non-pharmacological techniques are readily available to alleviate the distress of women in labour, the majority of women are unaware of its benefits. The objective of the study was to explore the impact of a simple non-pharmacological technique i.e., antepartum breathing exercises on maternal outcomes of labour among primigravid women.

Methods

A single centre prospective, single-blinded, randomized controlled trial was conducted at the antenatal outpatient clinic of a secondary healthcare institution. Eligible primigravid women were randomized into intervention and standard care groups. Both groups received standard obstetrical care. In addition, the intervention group were taught antepartum breathing exercises and were advised to practise daily and also during the active stage of labour. The primary outcome of the trial was the maternal outcome of labour measured in terms of onset of labour, nature of delivery, duration of labour, and need for augmentation of labour. Data was collected using World Health Organization (WHO) partograph, structured observational record on the outcome of labour.

Results

A total of 98 (70%) primigravid women who practised antepartum breathing exercises had spontaneous onset of labour. The odds of spontaneous onset of labour after randomization in the intervention group was 2.192 times more when compared to standard care at a (95% confidence interval 1.31–3.36, *p*<.001). Also, the requirement for augmentation of labour was minimal and there was a reduction in the rate of caesarean deliveries ( *p* <.05) based on the χ2 test. The overall mean duration of labour was less compared to standard care group F(1)= 133.800, *p* <.001.

Conclusion

Antepartum breathing exercises during labour can facilitate spontaneous vaginal birth, shorten the duration of labour, and reduce the need for operative interference.

## Introduction

The health and well-being of mother and child during pregnancy and childbirth are public health concerns because both of them have special needs, which cannot be catered to by general health services. ^1^ Prompt continuum of antenatal care is a significant maternal service that a woman needs to receive during pregnancy to have an optimal health outcome, facilitate timely treatment and prepare for childbirth.

Childbirth is a life-transforming intense event to a woman and her family. ^2^ It is the time to maintain balance between fulfilling experience and reality of expectations. Although childbirth is highly considered as one of the medical concern, yet lot of emphasis is given to natural birth with minimal intervention. Therefore, this becomes an ideal time to advise women on the normal physiological processes of pregnancy and childbirth that would help them to be prepared. The birthing environment greatly influences the experience during labour. ^3^ Fear of childbirth, lack of professional and family support during labour makes the birthing centres restrictive, at the same time if women is empowered with planned prenatal education to childbirth options such as positions, pain relief choices, use of relaxation techniques limits the routine use of medications, intravenous fluids, continuous electronic fetus monitoring, and other procedures carried out during labour.

A systematic review on childbirth education reports that childbirth preparation helps in building self-esteem, self-confidence and control, but weakly validates impact on reducing interventions during labour. ^4^ Non-pharmacological interventions/techniques or complementary therapies integrated into the routine antenatal childbirth education framework offers remarkable benefits to women during pregnancy and labour to have a satisfying labour experience with minimum labour interventions. ^5^

A variety of non-pharmacological techniques such as relaxation, breathing techniques, positioning/movement, massage, hydrotherapy, hot/cold therapy, music, guided imagery, acupressure, and aromatherapy are available to women in labour. Women are encouraged to employ any of these techniques, as they are non-invasive and appear to be safe for mother and baby.

Breathing is one of the simple, cost-effective non-pharmacological techniques, which connects the mind and the body, combination of controlled breathing and conscious relaxation are power-packed tools for labour. ^6^ Breathing distracts the focus away from pain and enables the mother-to-be to give birth awake and aware. ^7^ It also enables the woman to control her response to labour ^8^ and adjust her breathing levels as labour progresses. ^9^

A clinical trial conducted on pregnant women to assess the efficacy of supervised antenatal yoga program, which included breathing patterns on perceived maternal labour pain and birth outcomes reported that women experienced lower pain intensity, required decreased need for labour induction, showed lower rates of caesarean deliveries and experienced a shorter duration of second as well as the third stage of labour. ^10^

The Cochrane Systematic Review on the effectiveness of the non-pharmacological intervention on pain management for labour, reports relaxation, massage acupuncture, and hydrotherapy helped in the management of labour with few side effects; however, more exploration is needed to establish the efficacy of these techniques. ^11^

In response to the need to establish a strong evidence base for non-pharmacological intervention, we undertook a randomized controlled trial to test the hypothesis that women who participated in an antepartum breathing exercise program, in addition to usual antenatal care, would experience the spontaneous onset of true labour, spontaneous vaginal birth, shorter duration of labour, and lesser demand for augmentation of labour than antenatal women who receive standard antenatal care alone.

## Methods

### Study design

The antepartum breathing exercise program was based on the Lamaze method, with the Lamaze breathing component for use during pregnancy and childbirth. Antenatal women were recruited to a two-arm study consisting of an intervention group, who received training of the antepartum breathing exercise program in addition to usual care, and a standard care group, who received standard care alone. The study was a single-blinded and prospective randomized controlled trial (RCT). This trial was developed based on the extension of the CONSORT statement for reporting RCT. ^12^

### Ethical considerations

Ethical clearance was obtained from the Institutional Ethics Committee of Kasturba Hospital, Manipal (IEC 212/2012) and the trial was registered under The Clinical Trials Registry - India (CTRI). The registration number for this trial was CTRI/2016/02/006621, registered on 05.02.2016 (Available at URL http://www.ctri.nic.in).

### Participants

Women attending the antenatal clinic were eligible to participate in the study from 36 weeks of gestation. They were provided with a subject information sheet. Those women who were willing, interested, and eligible to participate, signed individual consent forms. Women were eligible to enter the trial if they had a singleton pregnancy with a cephalic presentation, had low risk (no pre-existing medical complications or existing obstetric complications), and were first-time childbearing women (primigravida). Women were excluded from entering the trial if they had pre-identified risk factors like eclampsia, preterm labour, placenta previa, multiple gestation, malpresentation and malposition or had been previously randomized to the trial. Recruitment was undertaken at one tertiary health care hospital in Udupi, Karnataka. All eligible antenatal women were approached in the antenatal clinic individually and were randomized to the study.

### Randomization

We used block randomization to randomize participants into the groups. Randomization was done in a 1:1 allocation ratio to ensure equal numbers in each group. Further allocation of participants to the intervention and standard care groups was done by an outpatient nurse with the help of a sequentially numbered opaque sealed envelope (SNOSE). The randomization of the study is illustrated in the reporting guidelines [ref https://doi.org/10.6084/m9.figshare.19076597.v1].

### Intervention

**Figure 1.**
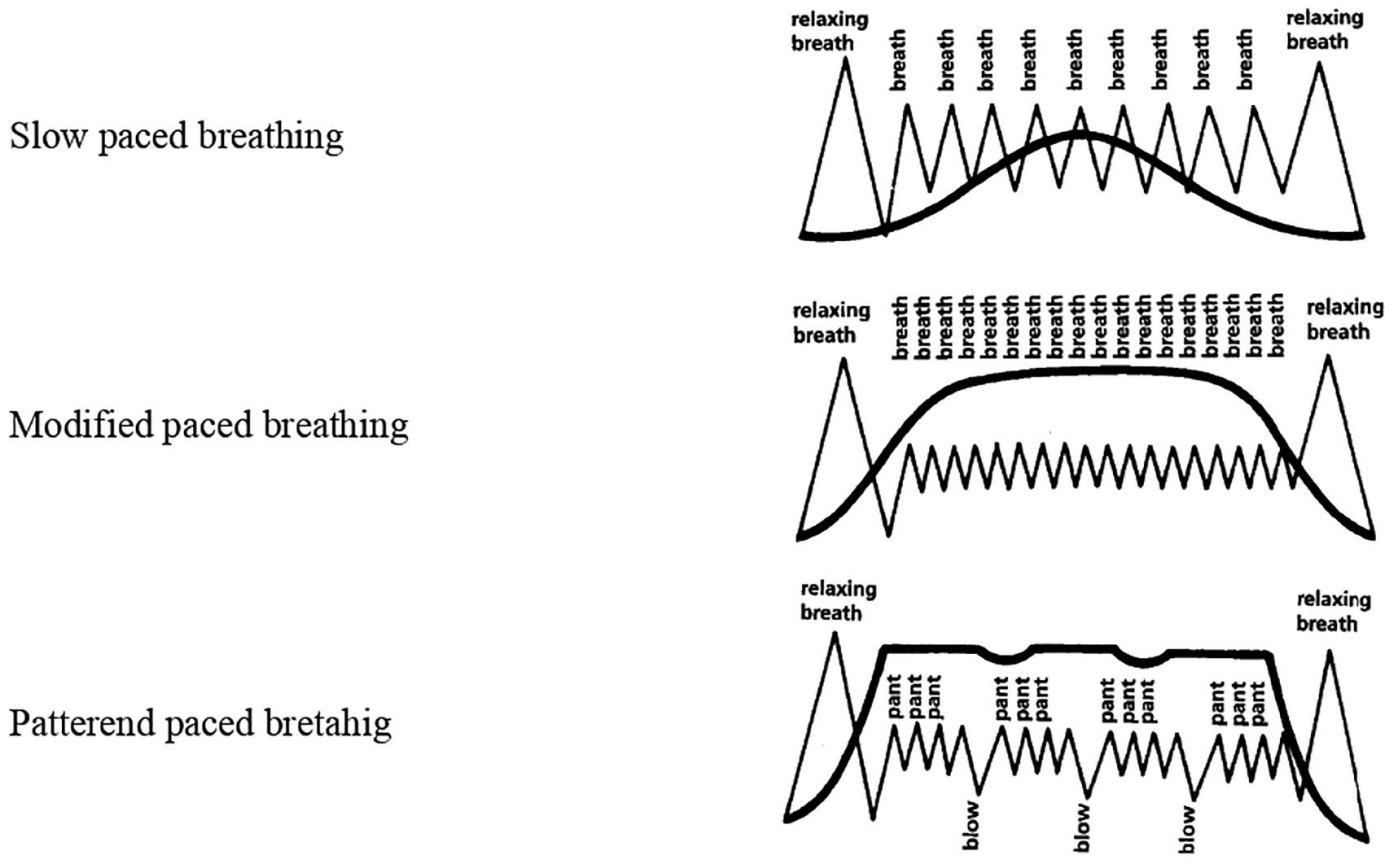
Images of Lambze breathing patterns.

Five breathing patterns were introduced namely- cleansing breathing for relaxation, slow-paced breathing, modified-paced breathing and patterned-paced breathing. These patterns were used during and following contractions. Gentle pushing, and breath-hold during pushing were instructed during the second stage of labour which encouraged descent of the baby ( Figure 1). The pattern of intervention was as follows:

*At 36 weeks of gestation*

An educational video explaining five patterns of breathing exercises was shown and their benefits were taught to women enrolled in the intervention group during the first appointment. These breathing patterns were demonstrated by the investigator to the women on a one-to-one basis. Women were asked to repeat these breathing patterns immediately after teaching and were advised to practice them twice daily for 15 minutes. Instructions were given to continue during the active phase of the first stage of labour under the supervision of labour room nurses.

*At 37– 40 weeks*

Women in the intervention group continued practising breathing exercises and compliance was monitored with help of a daily log along with the daily fetal movement count (Sadovsky method, as advised by obstetrician). This daily log was followed-up by the investigator during weekly antenatal visits. Occasionally the investigator checked for compliance through phone calls and enquired if they had any difficulties.

*During labour*

Monitoring during labour was done at the active phase of labour by the observers (nurses in the labour room), who were oriented on breathing exercises.

*Standard care*

Likewise, the women randomized to the standard care group received health talk on antepartum care and services according to local health care provision. They were also monitored during the active phase of labour by the observers in the labour room.

### Validity, reliability and rigour

Maternal and neonatal outcomes were measured using structured observational records on outcomes of labour. The tool consisted of 25 items that observed outcomes, which were the outcome of labour, nature of delivery, duration of labour, rate of episiotomy, augmentation of labour, gestational age at birth, birth injuries, and APGAR score at birth. The tool shows an accepted validity with an inter-rater reliability of one. Observational checklist on the performance of antepartum breathing patterns during the active phase of labour had 24 items; was reliable with an inter-rater reliability of .92, and .89 for test-retest reliability.

### Sample size and power

The sample size calculation was based on two independent means derived from the pilot study. The trial was designed to demonstrate minimum detectable differences in the duration of labour between two groups as one hour and anticipate an attrition rate of 10% since outcomes were measured until 40 weeks of gestation. This required a total sample size of 140 primigravid women in each arm of the trial for 80% power at a significance level of p<.05. Recruitment continued until at least 140 women had been enrolled, and from those randomized to the intervention group, 138 completed the study and two were excluded due to elective Lower Segment Caesarean Section (LSCS) or non-reactive Non Stress Test (NST). A low dropout rate (<7%) was observed for the overall study population.

### Data collection

The demographic data included the background information of the women’s age, period of gestation, last menstrual period (LMP), expected date of delivery (EDD), parity, years of marriage, religion, type of family, education, occupation, family income, height, current weight, and total weight gained during pregnancy. Maternal and neonatal outcomes were measured using structured observational records on the outcome of labour. This tool consisted of 12 items; maternal outcome included the onset of labour, duration of labour, the rate of episiotomy, and mode of delivery. Gestational age at birth, birth weight, birth injuries, APGAR score, and presence/absence of birth injuries were the parameters for neonatal outcomes. The performance of antepartum breathing exercises by the women during the active phase of labour was observed by the labour room nurses using an observation checklist. This tool included a stepwise performance of breathing patterns with ‘yes’ or ‘no’ options. If the women followed the steps, a score of ‘1’was given and if they failed to demonstrate, a score of ‘0’ was given.

### Data analysis

Categorical data were expressed as frequency and percentages and analyzed with the χ ^2^ test and Fisher's exact test. All continuous variables were expressed as the mean with standard deviation and analyzed using a two-way ANOVA test to compare the interaction between the independent variable on the dependent variable. Logistic regression analysis was performed to determine the independent predictive factors for spontaneous onset of labour. The data collected was analyzed using Statistical Package for Social Sciences (SPSS) version 17.0 SPSS (RRID:SCR_002865), URL: http://www-01.ibm.com/software/uk/analytics/spss/, significance was set at an α of 0.05, reporting on relative risk with a 95% CI.

## Results

### Participant characteristics

Out of 290 participants recruited, 280 participants were randomized into intervention or standard care groups. Nineteen women (2 from intervention group and 17 from the standard care group) were excluded from the study because they had elective caesarean deliveries and non-reassuring NST. Ultimately, 261 participants were enrolled with 138 in intervention group and 123 in standard care groups as shown in CONSORT flow diagram (Figure 1). The mean age was 26.51 (SD 2.88) years. The majority (92%) were married for 1-3 years. Most (66%) belonged to a joint family. Two-thirds (64%) were homemakers. The mean period of gestation at the time of recruitment for the study was 36.76 (SD 0.83) weeks. Since no significant differences were found, the groups were homogenous concerning demographic characteristics at the time of recruitment to the study ( Table 1).

**Table 1.** Baseline characteristics of participants.

Variables	Intervention (n=140)		Standard care (n=140)		*p-* value
	f	%	f	%	
**Years of marriage**					
1-3 years	**132**	**94**	**120**	**86**	.40
4-9 years	08	06	18	13	
Above 10 years	0	0	02	01	
**Type of family**					
Nuclear	47	34	57	41	.464
Joint	**93**	**66**	**83**	**59**	
**Occupation**					
Professional	31	22	11	08	.115
Unskilled	02	01	01	01	
Self employed	18	13	23	16	
Homemakers	**89**	**64**	**105**	**75**	
**Medical disorders during pregnancy**					
No	**126**	**90**	**119**	**85**	.798
Yes	14	10	21	15	

	Mean (SD)	Mean (SD)	
Mean age (in years)	26.51 ± 2.883	26.75 ± 3.595	.362
Period of gestation at enrolment	36.76 ± 0.830	36.83 ± 0.873	.191
Mean Hb (mg/dL)	12.07 ± 1.080	13.00 ± 0.094	.448
Mean weight gain (in kg)	10.85 ± 3.776	11.28 ± 3.683	.543
Mean weight gain after 36 weeks (in kg)	1.3 (1.1)	1.4 (1.04)	.526

Chi-square test was used for comparison of the proportion of categorical data including years of marriage, religion, type of family, occupation, medical disorders during pregnancy. One way ANOVA was used for comparison of continuous data including age, period of gestation, haemoglobin, and weight gain.

#### Primary outcome

*Onset of labour*

A statistically and clinically significant difference was found in the onset of true labour in our study. Among 138 primigravid women in the intervention group, 98 (70%) had spontaneous onset of labour (women came with term gestation, vertex presentation and spontaneous uterine contractions with or without rupture of membranes) as compared to those women in the standard care group. The logistic regression analysis ( Table 2) showed that odds of spontaneous onset of labour in the intervention group was 2.192 times more when compared to the standard care group with a 95% confidence interval (1.31–3.36) *p* <.001.

**Table 2.** Onset of labour in the intervention and standard care group.

Onset of labour	Intervention (N=138)	Standard care (N=123)	OR (95% CI)	*p-*value
Spontaneous	98 (70%)	66 (54%)	2.192 (1.313-3.361)	<.001
Non-spontaneous	40 (30%)	57 (46%)		

*p*<.05.

*Nature of delivery*

Women in the intervention group 67 (48%) were more likely to experience a spontaneous vaginal birth, 33% of women had induced vaginal deliveries and 19% had caesarean deliveries ( Table 3). The rate of spontaneous vaginal delivery was significantly lower and caesarean delivery was higher in the standard care group.

**Table 3.** Nature of delivery in the intervention and standard care group.

Nature of delivery	Intervention (N=138)	Standard care (N=123)	*p*-value
Spontaneous vaginal delivery	**67 (48%)**	17 (14%)	<.001
Induced delivery	45 (33%)	46 (37%)	
Caesarean deliveries	**26 (19%)**	60 (49%)	

The *p*-values were based on the λ ^2^ test, *p*<.05.

*Duration of labour*

**Figure 2.**
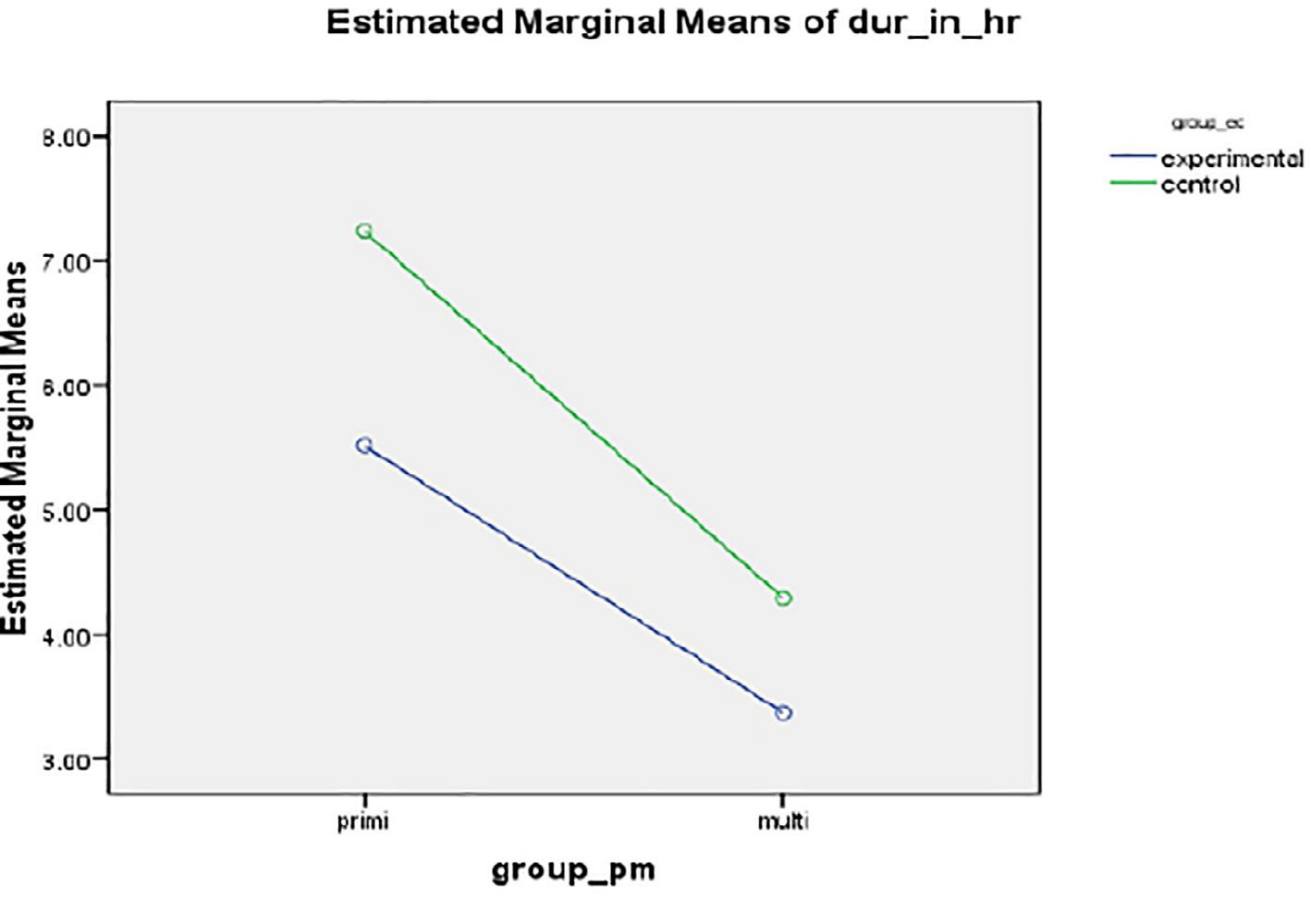
Mean profile plot showing duration of labor between intervention and standard care group.

A statistically and clinically significant difference was found in the mean duration of labour (in hours) between intervention 5.5127 (SD 1.998) hours and standard care group 7.238 ± 3.678 hours, resulting in a mean of 132 minutes, *p*<.001 ( Figure 2).

### Secondary outcome

Among 98 women in the intervention group who had spontaneous onset of labour, 68% had a spontaneous vaginal delivery with episiotomy, 18% had assisted vaginal delivery with outlet forceps due to failure of maternal power, decreased bearing down efforts and prolonged second stage of labour and 13% had vacuum delivery. Whereas in the standard care group, 26% had a spontaneous vaginal delivery, 34% had forceps and 40% had vacuum vaginal delivery (Data not shown).

## Discussion

Findings from our study suggested that breathing patterns when done regularly during the antenatal period as well as the active phase of labour had a positive impact on outcomes of labour. Excessive interventions at the time of labour were minimized preserving autonomy and ultimately enabling women to attain self-control. The biological characteristics in the study such as age and period of gestation were consistent with the findings of the earlier reports. ^13^

The benefits of control over maternal weight gain during pregnancy seen in our study supports the findings of study by Narendran S, *et al.,* suggesting that practicing yoga could prevent excessive weight gain and obesity during pregnancy. ^13^ Supervised exercise of light to moderate intensity can be used to prevent excessive gestational weight gain, especially in normal-weight women. ^14^

The most convincing reason to let labour begin on its own is the activation and stimulation of hormones like oxytocin, endorphins, catecholamines, and prolactin which regulate labour and birth. ^15^ The present study adds to the evidence that the odds of a natural onset of labour in the intervention group was 2.192 times more when compared to the standard care group with a 95% confidence interval [1.31-3.36], *p*<.001. Various other studies have shown that beta-endorphin levels were high after exercise ( *p* <.001), which is necessary for the spontaneous onset of labour, ^16^ and exercise during labour excited uterine contraction. ^17^

Breathing exercises during pregnancy revealed various beneficial effects like reduction in preterm birth, longer gestational age, increase in birth weight, reduction in the rate of caesarean birth and assisted vaginal birth. ^18^ The current study revealed that most women - almost 48% had a spontaneous vaginal birth and only 33% required the augmentation of labour with oxytocin in the intervention group. Supporting the findings of our study, a RCT carried out by Bergstro *et al.,* reports that among the women who were taught psychoprophylaxis method (including breathing exercises) 321 (66%) had spontaneous vaginal birth, 67 (14%) had instrumental birth and 96 (20%) had caesarean birth. ^19^ A similar result was reported in a systematic review by Curtis *et al.* (2012). ^20^ A study by Karkada *et al.* (2017) reported that women who had spontaneous onset of labour had 1.93 odds (95% CI, 1.30-2.86, *p*<.001) of having a spontaneous vaginal birth. ^21^

Labour is a multidimensional physiological event that involves the balance of hormonal factors. ^22^ If these hormones are systematically promoted, supported, and protected throughout pregnancy to birth, it would help to enhance wellbeing and empower childbirth transition. The present study indicated that the mean duration of labour in the intervention group was 5.5127 (SD 1.998) hours and the standard care group was 7.238 (SD 3.678) hours, consistent with previous studies. ^23^^,^^24^

### Limitations

First, the participants were low-risk pregnant women above 36 weeks of gestation without any major complications and thus the study did not focus on the high-risk pregnancy population. Second, as it was a single-blinded study, the researcher was aware of the group assignment. Hence, a reporting bias may have occurred. Third, generalizability was limited as participants were recruited from a single centre and recruitment from many centres is needed to replicate the findings.

### Implications for practice

The study provides policymakers with the evidence of incorporating comprehensive childbirth education that introduces women to a variety of options, which is not commonly practiced in India, especially in a setting where research was carried out. Instituting midwifery-led collaborative care services in community health centres and rural centres will aid in the utilization of these strategies and thus be sustainable. Secondly, a combination of evidence-based practice framework with an active-learning approach supports the development of an educational intervention that is intended to bring about a change in practice and meet the learning needs of labour and delivery.

## Conclusion

Considering the special needs of childbearing women, a personalized and focused protocol is best indicated, that adapts to a variety of practices, and which provides and promotes a holistic approach to health. It must also fosters participants with a framework with which they can integrate this practice during pregnancy and childbirth. Evidence from various studies and the present study support that antepartum breathing exercises are suitable for pregnancy and have an optimistic outcome. To add strength to the study, more standardized programs along with obstetricians should be conducted, which would bring improvements to evidence-based evaluations in a research environment.

## Data availability

### Underlying data

OSFHOME: Underlying data for “Effectiveness of antepartum breathing exercises on the outcome of labour: A randomized controlled trial”, https://doi.org/10.17605/OSF.IO/32GCV. ^25^

This project contains the following underlying data:

- Deidentified demographic information
- Deidentified comparison scores of primary outcomes between standard care and intervention group.

Data are available under the terms of the Creative Commons Zero “No rights reserved” data waiver (CC0 1.0 Public domain dedication).

### Reporting guidelines

Figshare: CONSORT checklist for‘[Effectiveness of antepartum breathing exercises on the outcome of labour: A randomized controlled trial]’. https://doi.org/10.6084/m9.figshare.19074524.v1

[CONSORT flowchart] https://doi.org/10.6084/m9.figshare.19076597.v1

Data are available under the terms of the Creative Commons Zero “No rights reserved” data waiver (CC0 1.0 Public domain dedication).

## Author contribution

**Table:** 

Author	Contribution
SRK	Conceptualization, Investigation, Methodology, Project administration, writing original
JAN	Conceptualization, Methodology, Supervision, Validation, Visualization, Writing-review
PB	Fund acquisition, Supervision, Validation, Visualization, Resources
SKB	Methodology, Supervision, Visualization, Writing-review, Resources
BSN	Supervision, Validation, Visualization, Writing-review
