# Supplementary material for: Effectiveness of antepartum breathing exercises on the outcome of labour: A randomized controlled trial
Source: F1000Res. 2023 May 30;11:159. Originally published 2022 Feb 8. [Version 3] doi: 10.12688/f1000research.75960.3 (PMC10359740; doi:10.12688/f1000research.75960.3)
Supplement: Peer review history

# Peer review: Reviewer response for version 3

**DOI:** 10.5256/f1000research.149191.r175887

**Published:** 2023-07-20

**Authors:** Udara Dilrukshi Senarathne

**Affiliations:** 1 Department of Biochemistry, Faculty of Medical Sciences, University of Sri Jayewardenepura, Nugegoda, Sri Lanka; 2 Pathology, Monash Health, Clayton, Victoria, Australia

The authors have changed the context of the questionable section providing general reasoning which would be prohibitive to the routine practice of labor care without giving references, which would not affect the validity of the study's findings.

Is the work clearly and accurately presented and does it cite the current literature?

Yes

If applicable, is the statistical analysis and its interpretation appropriate?

Yes

Are all the source data underlying the results available to ensure full reproducibility?

Yes

Is the study design appropriate and is the work technically sound?

Yes

Are the conclusions drawn adequately supported by the results?

Yes

Are sufficient details of methods and analysis provided to allow replication by others?

Yes

Reviewer Expertise:

Pathology, inborn errors of metabolism, early neonatal care, gender based violence

I confirm that I have read this submission and believe that I have an appropriate level of expertise to confirm that it is of an acceptable scientific standard.

# Peer review: Reviewer response for version 2

**DOI:** 10.5256/f1000research.140102.r170675

**Published:** 2023-05-25

**Authors:** Udara Dilrukshi Senarathne

**Affiliations:** 1 Department of Biochemistry, Faculty of Medical Sciences, University of Sri Jayewardenepura, Nugegoda, Sri Lanka; 2 Pathology, Monash Health, Clayton, Victoria, Australia

The manuscript discusses about the antepartum breathing exercises on maternal outcomes of labour among primigravid women in the form of a randomised controlled trial. The authors have given training on antepartum breathing exercises to be practiced during antepartum period and the active stage of labour to the intervention group and the primary outcome had been assessed based on maternal outcome of labour in terms of onset of labour, nature of delivery, duration of labour, and need for augmentation. The manuscript reads effortlessly and clearly points out the major facts. The abstract provides a clear understanding to the readers of the content.

- The introduction is well written to lay the basis for the intended study supported by available evidence.
- Suggest rewording the following sentence:
*"Most of the birthing centres in the country are restrictive with routine use of medications, intravenous fluids, continuous electronic fetus monitoring, and numerous procedures that are carried out as part of typical labour."*
- Briefly indicate what factors (e.g: X, Y, & Z) may prevent the routine use of above-mentioned treatments in India with references as this creates the basis for application of the suggested intervention.
  E.g.: X, Y, & Z can be prohibitive to the routine use of medications, intravenous fluids, continuous electronic foetus monitoring, and other procedures carried out during labour in most birth centres in India.
- The methodology is written including an adequate description of the methods to allow recreation of the study following CONSORT guidance.
- The interventions are well-described and appropriately executed. 
- Suggest making the educational video on breathing exercises available as supplementary material if possible.
- Robust statistical analysis has been applied to analyse data and results are presented appropriately.
- The authors correlate the current findings with existing evidence to come to a conclusion indicating the statistically significant outcome with the suggested intervention.
- The application of breathing exercises in labour is a good and feasible practice to be introduced in low-resource settings. This study provides statistically significant evidence supporting its applications.

Is the work clearly and accurately presented and does it cite the current literature?

Yes

If applicable, is the statistical analysis and its interpretation appropriate?

Yes

Are all the source data underlying the results available to ensure full reproducibility?

Yes

Is the study design appropriate and is the work technically sound?

Yes

Are the conclusions drawn adequately supported by the results?

Yes

Are sufficient details of methods and analysis provided to allow replication by others?

Yes

Reviewer Expertise:

Pathology, inborn errors of metabolism, early neonatal care, gender based violence

I confirm that I have read this submission and believe that I have an appropriate level of expertise to confirm that it is of an acceptable scientific standard, however I have significant reservations, as outlined above.

# Peer review: Reviewer response for version 2

**DOI:** 10.5256/f1000research.140102.r154860

**Published:** 2022-11-04

**Authors:** Lily Podder, Anurag Bhai Patidar

**Affiliations:** 1 Department of Obstetrics and Gynecological Nursing, Nursing College, All India Institute of Medical Sciences Bhopal, Bhopal, Madhya Pradesh, India; 2 All India Institute of Medical Sciences, Bhopal, Madhya Pradesh, India

I am satisfied with the correction and explanations incorporated to the review comments/suggestions.

Is the work clearly and accurately presented and does it cite the current literature?

Yes

If applicable, is the statistical analysis and its interpretation appropriate?

Yes

Are all the source data underlying the results available to ensure full reproducibility?

Yes

Is the study design appropriate and is the work technically sound?

Partly

Are the conclusions drawn adequately supported by the results?

Yes

Are sufficient details of methods and analysis provided to allow replication by others?

Yes

Reviewer Expertise:

NA

We confirm that we have read this submission and believe that we have an appropriate level of expertise to confirm that it is of an acceptable scientific standard.

# Peer review: Reviewer response for version 1

**DOI:** 10.5256/f1000research.79907.r149155

**Published:** 2022-09-09

**Authors:** Lily Podder, Anurag Bhai Patidar

**Affiliations:** 1 Department of Obstetrics and Gynecological Nursing, Nursing College, All India Institute of Medical Sciences Bhopal, Bhopal, Madhya Pradesh, India; 2 All India Institute of Medical Sciences, Bhopal, Madhya Pradesh, India

The writing style of the manuscript is very captivating. The guidelines of writing manuscripts are appropriately followed. The grant information is delineated adequately. The abstract of the manuscript is written in a crisp and clear manner. Introduction incorporates scientific evidences depicting the current scenario and need of the study.

 Research methodology is written adequately with relevant explanations and covered all the important elements of planning and conducting research. However, participants’ information is not matched with the CONSORT flowchart (290) with the information available at Pg no: 5 (292) of the manuscript under participants characteristics heading. Clarification is also required about randomizing the samples of 280 from recruited samples of 292. Explanation is also required for reason/s for excluding 09 participants while assessing for eligibility.

 The researcher crafted intervention is very extensively planned and implemented. The researcher is competent in providing the crafted interventions with added credentials.

 Analysis and interpretation of the data are well organized as per the research objectives. Extensive and Exhaustive analysis of the data done and presented with robust statistical operations.

 Summary, discussion, implications, conclusion of the research is presented and discussed in accordance with the findings of the present study.

Is the work clearly and accurately presented and does it cite the current literature?

Yes

If applicable, is the statistical analysis and its interpretation appropriate?

Yes

Are all the source data underlying the results available to ensure full reproducibility?

Yes

Is the study design appropriate and is the work technically sound?

Partly

Are the conclusions drawn adequately supported by the results?

Yes

Are sufficient details of methods and analysis provided to allow replication by others?

Yes

Reviewer Expertise:

NA

We confirm that we have read this submission and believe that we have an appropriate level of expertise to confirm that it is of an acceptable scientific standard, however we have significant reservations, as outlined above.

# Response

**Published:** 2022-09-10

**Authors:** SUSHMITHA KARKADA

**Affiliations:** Manipal College of Nursing, Manipal Academy of Higher Education, India

Dear Dr Lily Podder,

 I appreciate the time and effort that you have dedicated to provide your valuable feedback on my manuscript. Here are my clarifications for the comments: 

1. Will correct the error in Table 1, as the total samples enrolled based on eligibility criteria was 290.
2. Based on the sample size calculation, 140 samples to be recruited in each arm (140x2), thus 280 samples were selected through block randomization.
3. Nine samples were excluded as they opted government hospital for delivery. 

Look forward to hearing from you regarding the submission and to respond to any further questions and comments you may have.
